# A characterization of nonemptiness and boundedness of the solution set for set-valued vector equilibrium problems via scalarization and stability results

**DOI:** 10.1186/s40064-016-3001-z

**Published:** 2016-08-12

**Authors:** Pakkapon Preechasilp, Rabian Wangkeeree

**Affiliations:** 1 Program in Mathematics, Faculty of Education, Pibulsongkram Rajabhat University, Phitsanulok, 65000 Thailand; 2 Department of Mathematics, Faculty of Science, Naresuan University, Phitsanulok, 65000 Thailand

**Keywords:** Equilibrium problem, Barrier cone, Pseudomonotone mappings, Stability analysis

## Abstract

In this paper, the existence theorems of solutions for generalized weak vector equilibrium problems are developed in real reflexive Banach spaces. Based on recession method and scalarization technique, we derive a characterization of nonemptiness and boundedness of solution set for generalized weak vector equilibrium problems. Moreover, Painlevé–Kuratowski upper convergence of solution set is also discussed as an application, when both the objective mapping and the constraint set are perturbed by difference parameters.

## Background

Let *X* be a real reflexive Banach space and *Y* be a real normed linear space. Suppose that, and $C \subseteq Y$ is a nonempty, closed and convex pointed cone with $int \ C \ne \varnothing$. Let $K \subseteq X$ be a non-empty subset and a set-valued function $F : K \times K \rightarrow 2^Y\backslash \{\varnothing \}$, the following generalized weak vector equilibrium problem (GWVEP) is to find $\bar{x} \in K$ such that

GWVEP $$\begin{aligned} \bar{x}\in K \text { such that } F(\bar{x},y)\cap (-int \ C) = \varnothing , \ \forall y\in K, \end{aligned}$$

and the dual problem for (GWVEP), is so called (DGWVEP), is to find $\bar{x} \in K$ such that

DGWVEP $$\begin{aligned} \bar{x}\in K \text { such that } F(y,\bar{x})\cap (int \ C) = \varnothing , \ \forall y\in K. \end{aligned}$$

Both (GWVEP) and (DGWVEP) have been extensively studied by many authors (see Ansari and Flores-Bazán 2006; Ansari et al. 2001a, b, 2002; Flores-Bazán and Flores-Bazán 2003; Ansari et al. 2001; Sadeqi and Alizadeh 2011; Zhong et al. 2011). An important and interesting topic for (GWVEP) and (DGWVEP) is to study the nonemptiness and boundedness of the solution sets. As far as we known, the first paper which discussed this issues was Flores-Bazán and Flores-Bazán (2003) in the case where *F* is vector-valued. They studied the existence of solutions of (GWVEP) under the asymptotic analysis, where neither compactness of *K* nor any coercivity condition is assumed in reflexive Banach spaces. By using idea of recession method in Flores-Bazán and Flores-Bazán (2003), Ansari and Flores-Bazán (2006) gave some necessary and sufficient conditions for nonemptiness and boundedness of the solution set of (GWVEP). In 2011, Sadeqi and Alizadeh (2011) discussed and improved some results of Ansari and Flores-Bazán (2006). They gave the conditions under which the solution set of (GWVEP) is non-empty, convex and weakly compact subset in reflexive Banach spaces. After a thorough review of the literature and according to our knowledge, we found that the convexity assumed for second variable of *F* is an essential assumption (see also Chen et al. 2008; Flores-Bazán 2000; Fang and Huang 2007).

On the other hand, the stability analysis of the solution mappings to generalized vector equilibrium problem is an important topic in vector optimization theory. Recently, the lower semicontinuity, (Hölder) continuity of the solution maps to (GWVEP) are discussed in Li and Li (2011), Gong (2008), Chen et al. (2009), Xu and Li (2013). Among those papers, we observe that the linear scalarization technique is one effective to deal with the lower semicontinuity and (Hölder) continuity of solution mappings to (GWVEP). Based on the linear scalarization, the solution sets for (GWVEP) is the union of family of the solution set to scalarized equilibrium problems with respect to the linear map on dual cone. In natural, the union of family of solution sets to scalarized equilibrium problems is finer than the solution set to (GWVEP). In order to obtain the equality, convexity in second variable of *F* is assumed.

Motivated and Inspired by above works, the aim of this paper is to consider a (GWVEP) with a set-valued map on unbounded constraint set in reflexive Banach spaces. We first collect the characterization results of the nonemptiness and boundedness of the solution set of (GWVEP). By using the linear scalarization technique, we characterize the nonemptiness and boundedness of the solution set of (GWVEP) in terms of nonemptiness and boundedness of a family of scalar equilibrium problem with respect to linear maps in connected base for dual cone of *C*. Finally, we give the stability results for the solution maps to (GWVEP) in the sense of Painlevé–Kuratowski upper convergence of solution set.

The paper is organized as follows. In “Preliminaries” section, we introduce some basic notations and preliminary results. In “Characterization of nonemptiness and boundedness of the solution set” section, by using a scalarization technique, we establish the nonemptiness and boundedness of solution set for (GWVEP) in reflexive Banach spaces. In “Stability analysis” section, we give an application to the stability of the solution sets for (GWVEP).

## Preliminaries

Throughout this paper, unless otherwise specified, we always assume that *X* is a real reflexive Banach space, *Y* is a real normed space with dual space $Y^*$ and $C\subseteq Y$ is a nonempty, closed, convex and pointed cone with $int \ C \ne \varnothing$. Let

$$\begin{aligned} C^* := \{\xi \in Y^* : \langle \xi , y\rangle \ge 0, \quad \forall y \in C\} \end{aligned}$$

be the dual cone of *C*. Clearly,

$$\begin{aligned} y\in C\Leftrightarrow & {} \langle \xi ,y \rangle \ge 0, \quad \forall \xi \in C^*, \\ y\in int\,C\Leftrightarrow & {} \langle \xi ,y \rangle > 0, \quad \forall \xi \in C^*. \end{aligned}$$

Since $int \, C\ne \varnothing$, for any fixed $e \in int \ C$, it proved in Huang et al. (2014) that the dual cone $C^*$ of *C* has a following weak $^*$ compact base $C^{*0}$.

$$\begin{aligned} C^{*0}:=\{\xi \in C^* :\langle \xi , e\rangle = 1\}, \end{aligned}$$

where a subset $D\subset C^*$ is said to be a base of $C^* \Leftrightarrow 0\notin D$ and $C^*\subset \cup _{t\ge 0} t D$.

A vector $x \in K$ is called weak efficient solution to the (GWVEP) if

1 $$\begin{aligned} F(x,y) \cap (-int \ C) = \varnothing , \quad \forall y \in K, \end{aligned}$$

and weak efficient solution to the (DGWVEP) if

2 $$\begin{aligned} F(y,x) \cap (int \ C) = \varnothing , \quad \forall y \in K. \end{aligned}$$

Denote by $S^P_W(K,F)$ and $S^D_W(K,F)$ the set of all weak efficient solution to the (GWVEP) and (DGWVEP), respectively.

### **Definition 1**

(Zhong et al. 2011) Let *K* be a non-empty convex subset of *X*. For a given closed convex cone *C* of a real normed space *Y* , the set-valued map $F : K \rightarrow 2^Y\backslash \{\varnothing \}$ is said to be

(i) upper *C*-convex, if for any $x,y\in K$and for any $t\in [0,1]$,
  $$\begin{aligned} tF(x) + (1-t)F(y) \subseteq F(tx + (1-t)y) + C; \end{aligned}$$
(ii) lower *C*-convex, if for any $x,y\in K$ and for any $t\in [0,1]$,
  $$\begin{aligned} F(tx + (1-t)y) \subseteq tF(x) + (1-t)F(y) - C; \end{aligned}$$
(iii) *C*-convex, if *F* is both upper *C*-convex and lower *C*-convex.

### *Remark 1*

If *F* is a upper *C*-convex map on *K*, then for any $x\in K , F(x) + C$ is convex set.

We first recall the well-known concept of monotone mapping for a real set-valued mapping.

### **Definition 2**

A bifunction $f : K \times K \rightarrow 2^\mathbb {R}\backslash \{\varnothing \}$ is said to be

(i) monotone on *K*, if for any $x,y\in K$
  $$\begin{aligned} z+ z' \le 0, \quad \forall z\in f(x,y),z'\in f(y,x); \end{aligned}$$
(ii) pseudomonotone on *K*, if for any $x,y\in K$
  $$\begin{aligned} z \ge 0, \quad \forall z\in f(x,y) \Rightarrow z' \le 0, \ \forall z'\in f(y,x). \end{aligned}$$

It is well-known that every monotone map is pseudomonotone map.

In the case where *F* is a vector set-valued, the concept of monotonicity can be also extended as follows.

### **Definition 3**

Let $C\subseteq Y$ be a nonempty, closed, convex and pointed cone with $int \ C \ne \varnothing$. A set-valued map $F : K \times K \rightarrow 2^Y\backslash \{\varnothing \}$ is said to be

(i) *C*-monotone if, for all $x, y \in K$,
  $$\begin{aligned} F(x,y) + F(y,x) \subseteq - C; \end{aligned}$$
(ii) *C*-pseudomonotone type *I* if, for all $x, y \in K$,
  $$\begin{aligned} F(x,y)\cap (-int \ C) = \varnothing \Rightarrow F(y,x) \cap (int \ C) = \varnothing ; \end{aligned}$$
(iii) *C*-pseudomonotone type *II* if, for all $x, y \in K$,
  $$\begin{aligned} F(x,y)\cap (-int \ C) = \varnothing \Rightarrow F(y,x) \subseteq - C; \end{aligned}$$
(iv) $$\xi $$-monotone w.r.t. $$C^{*} $$if, for any $$\xi \in C^{*} $$ and for any $$ x, y \in K $$,
  $$\begin{aligned} \xi (z) + \xi (z') \le 0, \quad \forall z\in F(x,y), \;\; \forall z'\in F(y,x); \end{aligned}$$
(v) $\xi$-pseudomonotone w.r.t. $C^{*}$ if, for any $\xi \in C^{*}$ and for any $x, y \in K$,
  $$\begin{aligned} \xi (z) \ge 0,\quad \forall z\in F(x,y) \Rightarrow \xi (z') \le 0, \quad \forall z'\in F(y,x). \end{aligned}$$

### *Remark 2*

1. It is clear that *C*-monotone mapping is *C*-pseudomonotone type *I* and type *II* and *C*-pseudomonotone type *II* implies *C*-pseudomonotone type *I*.
2. Every *C*-monotone mapping is $\xi$-pseudomonotone w.r.t. $C^{*}$.
3. Every *C*-pseudomonotone type *II* mapping is $\xi$-pseudomonotone w.r.t. $C^{*}$, Indeed, for any $\xi \in C^{*}$ and for any $x,y\in K$ satisfying $\xi (z) \ge 0$ for all $z\in F(x,y)$, we have $z\notin -int \ C$ and so $F(x,y) \cap (-int \ C) = \varnothing$. $F(y,x) \subseteq - C$ implies that $\xi (z') \le 0$ for all $z'\in F(y,x)$. But, *C*-pseudomonotone type *I* may not implies $\xi$-pseudomonotone w.r.t. $C^{*}$.

### *Example 1*

Let $X=\mathbb {R}, K=[0,1] , Y=\mathbb {R}^2 , C=\mathbb {R}^2_+$. Define $F:K\times K \rightarrow 2^Y\backslash \{\varnothing \}$ by

$$\begin{aligned} F(x,y) = {\left\{ \begin{array}{ll} (x,-x) &\quad {}\text { if } x=y, \\ \{(y-x)\}\times [0,(y-x)] &\quad {}\text { if } y-x>0, \\ \{(y-x)\}\times [(y-x),0] &\quad {}\text { if } y-x<0. \end{array}\right. } \end{aligned}$$

Thus, clearly that *F* is $\xi$-pseudomonotone on *K* w.r.t. $C^{*} \equiv C$. Indeed, for any $x,y\in K$ and $\xi \in C^{*}$ if $\xi (F(x,y)) \ge 0$, then $y-x> 0$. This implies that

$$\begin{aligned} F(y,x) = \{(x-y)\}\times [(x-y),0] \subseteq -\mathbb {R}_+^2 \Rightarrow \xi (z) \le 0, \quad \forall z\in F(y,x). \end{aligned}$$

But *C*-pseudomonotone type *II* in the case when $x=y$.

### *Example 2*

Let $X=\mathbb {R} , K=[0,+\infty ) , Y=\mathbb {R}^2 , C=\mathbb {R}^2_+$ and $C^*\equiv C$. Define $F:K\times K \rightarrow 2^Y\backslash \{\varnothing \}$ by

$$\begin{aligned} F(x,y) = \{0\}\times [0,|y-x|], \quad \forall x,y\in K. \end{aligned}$$

Thus, clearly that for any $x,y\in K , F(x,y)\cap (-int\,C) = \varnothing$ implies that $F(y,x)\cap (int\,C) = \varnothing$. Hence, *F* is pseudomonotne on *K* type *I*, but not *C*-pseudomonotone type *II*.

Moreover, for any $\xi \in C^{*}$ and $x,y\in K$, we then have

$$\begin{aligned} \xi \left( F(x,y)\right) = \xi \left( F(y,x)\right) \ge 0. \end{aligned}$$

Therefore, *F* is not $$ \xi $$-pseudomonotone on *K* w.r.t. $$C^{*} $$ as shown in the following example.

### **Definition 4**

A topological space *E* is said to be connected iff, it is not the union of two disjoint nonempty open sets. Moreover, *E* is said to be path-connected iff, any two points of *E* can be joined by a path.

The following lemma, which gives an equivalent characterization of connected spaces, plays an important role in our proof.

### **Lemma 1**

*A topological spaceEis connected if and only if the only subsets ofEwhich are both open and closed areEand*$\varnothing$.

### **Definition 5**

Let $F : K \rightarrow 2^Y$ be a set-valued mapping with nonempty values. *F* is said to be

(i) upper semicontinuous(u.s.c.) on *K* iff, for every $x \in K$ and every neighborhood *N*(*F*(*x*)) of *F*(*x*) , there exists a neighborhood *N*(*x*) of *x* such that $F(N(x)) \subseteq N(F(x))$;
(ii) lower semicontinuous(l.s.c.) on *K* iff, for every $x \in K , u \in F(x)$ and every neighborhood *N*(*u*) of *u*, there exists a neighborhood *N*(*x*) of *x* such that $F(x')\cap N(u) \ne \varnothing$ for every $x' \in N(x)$.

### **Proposition 1**

(Aubin and Ekeland 1984; Ferro 1989)

(i) *Fis l.s.c. at*$\bar{\lambda }$*if and only if for any sequence*$\{\lambda _n\} \subset \Lambda$*with*$\lambda _n\rightarrow \bar{\lambda }$*and any*$\bar{x}\in F(\bar{\lambda })$, *there exists*$x_n \in F(\lambda _n)$*such that*$x_n \rightarrow \bar{x}$.
(ii) *Fis weakly l.s.c. at*$\bar{\lambda }$*if and only if for any sequence*$\{\lambda _n\} \subset \Lambda$*with*$\lambda _n\rightharpoonup \bar{\lambda }$*and any*$\bar{x}\in F(\bar{\lambda })$, *there exists*$x_n \in F(\lambda _n)$*such that*$x_n \rightarrow \bar{x}$.
(iii) *IfFhas compact values (i.e.,*$F(\lambda )$*is a compact set for each*$\lambda \in \Lambda$), *thenFis u.s.c. at*$\bar{\lambda }$*if and only if for any sequence*$\{\lambda _n\}\subset \Lambda$*with*$\lambda _n\rightarrow \bar{\lambda }$*and for any*$x_n\in F(\lambda _n)$, *there exists*$\bar{x}\in F(\bar{\lambda })$*and a subsequence*$\{x_{n_k}\}$*of*$\{x_{n}\}$*such that*$x_{n_k}\rightarrow \bar{x}$.

We collect the following well-known KKM-Fan lemma.

### **Lemma 2**

(Fan 1984) *LetMbe a nonempty, closed and convex subset ofXand*$F : M \rightarrow 2^M\backslash \{\varnothing \}$*be a set-valued map. Suppose that for any finite set*$\{ x_1,\ldots , x_m \} \subseteq M$, *one has*

(i) $conv \{ x_1,\ldots ,x_m \} \subset \cup _{i=1}^m F(x_i )$ (*i.e., F is a KKM map on M*);
(ii) *F*(*x*) *is closed for every*$x \in M$; *and*
(iii) *F*(*x*) *compact for some*$x \in M$.

*Then*$$ \cap _{x\in M} F(x) \ne \varnothing $$.

Now, we recall the fundamental tools used throughout this paper. This leads to the concepts of asymptotic cone and asymptotic function through its epigraph.

$$\begin{aligned} K_\infty =\left\{ d\in X : \exists t_k\rightarrow + \infty , x_k\in X \text { such that }\dfrac{1}{t_k}x_k \rightharpoonup d \right\} , \end{aligned}$$

where “$\rightharpoonup$” or “$\omega -\lim \nolimits _{n\rightarrow \infty } x_n = x$” means convergence in the weak topology. In case *K* is convex subset, $K_\infty$ can also be determined by the following formula

$$\begin{aligned} K_\infty = \left\{ d\in X : x_0+ td \in K, \forall t>0, \quad \forall x_0\in K \right\} . \end{aligned}$$

The barrier cone of *K* is defined by

$$\begin{aligned} barr \ K = \left\{ \xi ^*\in K^* :\sup _{y\in K}\langle \xi ^*,y \rangle < +\infty \right\} . \end{aligned}$$

### **Proposition 2**

(Ansari and Flores-Bazán 2006, Proposition 2.1) *The following holds:*

(i) $$ K^1\subseteq K^2 $$*implies*$$ K^1_\infty \subseteq K^2_\infty $$;
(ii) $$ \left( K+x\right) _\infty = K_\infty , \ \forall x\in X $$;
(iii) *let*$\{K^i\}_{i\in I}$*be any family of nonempty sets inX* , *then*
  3 $$\begin{aligned} \left( \cap _{i\in I} K^i \right) _\infty \subset \cap _{i\in I} K^i_\infty . \end{aligned}$$

*If, in addition*, $\cap _{i\in I}K^i \ne \varnothing$*and each set*$K^i$*is closed and convex, then we obtain an equality in* (3).

### **Lemma 3**

(Adly et al. 2004) *Let Kbe a nonempty, closed and convex subset of a real reflexive Banach spaceXwith*$int(\text {barr } K) \ne \varnothing$. *Then there is no sequence*$\{x_n\} \subset K$*with*$\Vert x_n \Vert \rightarrow \infty$*such that origin is a weak limit of*$\dfrac{x_n}{\Vert x_n\Vert }$, *i.e.*$\dfrac{x_n}{\Vert x_n\Vert } \rightharpoonup 0$.

### **Lemma 4**

(Fan and Zhong 2008) *LetKbe a nonempty, closed, convex subset of a real reflexive Banach spaceXwith*$int\,(barr\,(K))\ne \varnothing$. *Then there exists no sequence*$\{d_n\}\subset K_\infty$*with each*$\Vert d_n\Vert =1$*such that*$d_n \rightharpoonup 0$.

### **Lemma 5**

(Fan and Zhong 2008) *Let* (*M*, *d*) *be a metric space and*$\mu _0\in M$*be a given point. Let*$K : M\rightarrow 2^X$*be a set-valued mapping with nonempty valued and upper semicontinuous at*$\mu _0$. *Then there exists a neighborhood*$N(\mu _0)$*of*$\mu _0$*such that*$(K(\mu )_\infty ) \subset (K(\mu _0))_\infty$*for all*$\mu \in N(\mu _0)$.

## Characterization of nonemptiness and boundedness of the solution set

In this section, we shall prove the characterization of nonemptiness and boundedness of the solution set for (GWVEP) which states that under suitable conditions.

First of all, we recall the existing assumptions and results which can be found in Ansari and Flores-Bazán (2006), Zhong et al. (2011), Sadeqi and Alizadeh (2011).

### **Assumption 1**

(Zhong et al. 2011; Ansari and Flores-Bazán 2006)* The set-valued map *$F:K\times K \rightarrow 2^Y\backslash \{\varnothing \}$* is such that: *

$$ (F_0) $$
$$ F(x,x) =\{0\} $$ for all $$ x\in K $$.
$$ (F_1) $$
*For any*$x,y\in K , F(x,y)\cap (-int \ C) = \varnothing \Rightarrow F(y,x) \subseteq -C$ (*C pseudomonotone type II ).*
$$ (F_2) $$
*For any *$x\in K , F(x,\cdot ):K\rightarrow 2^Y\backslash \{\varnothing \}$ is *C-convex.*
$$ (F_3) $$
*For any *$x,y\in K$*, the set*$\{ z\in [x,y] : F(z,y)\cap (-int \ C) = \varnothing \}$*is closed, where [x*, *y] denotes the closed line segment joining x and y* .
$$ (F_4) $$
*For any *$x\in K , F(x,\cdot )$* is weakly lower semicontinuous.*
$$ (F_5) $$
*For any *$y\in K , \{x\in K : F(y,x) \cap (int \ C) = \varnothing \}$* is convex.*

Under Assumption 1, It is proved in Zhong et al. (2011) that, $S^P_W(K,F)$ is nonempty if *K* is bounded subset of *X* . In the case where *K* is unbounded, it is needed to determine the behavior of *F* along some particular directions. We introduce the following cones.

4 $$\begin{aligned} R_{1} := \{ d\in K_\infty : F(y,y+td) \cap (int \ C) = \varnothing , \quad \forall y\in K, t>0 \}\text {{.}} \end{aligned}$$

The following lemma illustrates that the solution set $S^P_W(K,F)$ and $S^D_W(K,F)$ are coincide no matter what *K* is bounded or not.

### **Lemma 6**

(Sadeqi and Alizadeh 2011, Lemma 3.4)* Let K be a nonempty, closed and convex subset of X and *$F:K\times K \rightarrow 2^Y\backslash \{\varnothing \}$* be a set valued map satisfying * (*F*_0_)–(*F*_3_)*. Then*

$$\begin{aligned} S_W^P(K,F) = S_W^D(K,F). \end{aligned}$$

### **Theorem 1**

(Sadeqi and Alizadeh 2011, Theorem 3.5) *LetKbe a nonempty, closed and convex subset ofXand*$F:K\times K \rightarrow 2^Y\backslash \{\varnothing \}$*be a set valued map satisfying* (*F*_0_)–(*F*_5_). *If the set the solution set*$S^P_W(K,F)$*is nonempty, then*

$$\begin{aligned} (S^P_W(K,F))_\infty = (S^D_W(K,F))_\infty = R_1. \end{aligned}$$

The following theorem is due to the result in Zhong et al. (2011), Ansari and Flores-Bazán (2006), Sadeqi and Alizadeh (2011).

### **Theorem 2**

*LetKbe a nonempty closed convex subset ofXand*$F : K \times K \rightarrow 2^Y\backslash \{\varnothing \}$*be a set valued mapping satisfying assumptions* (*F*_0_)–(*F*_5_). *Suppose that*$int (barr(K)) \ne \varnothing$. *Then the following statements are equivalent*.

(i) *the solution set of*$S^P_W(K,F)$*is nonempty and bounded*;
(ii) *the solution set of*$S^D_W(K,F)$*is nonempty and bounded*;
(iii) $$ R_1=\{0\} $$;
(iv) *there exists a bounded set*$B \subset K$*such that for every*$x \in K \backslash B$, *there exists some*$y \in B$*such that*$F(y,x) \cap (int \ C) \ne \varnothing$.

### *Proof*

(i) $\Leftrightarrow$ (ii) and (ii) $\Rightarrow$ (iii) are obtained by Theorems 1 and 2, respectively.

(iii) $\Rightarrow$ (iv) Suppose not, if (iv) does not hold, then there exists a sequence $\{x_n\} \subseteq K$ such that for each $n , \Vert x_n\Vert > n$ and

$$\begin{aligned} F(y,x_n) \cap (int \ C) = \varnothing , \end{aligned}$$

for every $y \in K$ with $\Vert y\Vert \le n$. For fixed $y \in K$ and $t > 0$, without loss of generality, we may take a subsequence $\{x_{n_k}\}$ of $\{x_n\}$ such that

5 $$\begin{aligned} \dfrac{t}{\Vert x_{n_k} - y \Vert } \in (0,1)\quad \text { and }\;\; \ \omega -\lim _{k\rightarrow + \infty } \dfrac{x_{n_k} - y}{\Vert x_{n_k} - y \Vert } = \omega -\lim _{k\rightarrow + \infty } \dfrac{x_{n_k}}{\Vert x_{n_k} \Vert } = d_0 \in K_\infty . \end{aligned}$$

Thanks to Lemma 3, one has $d_0 \ne 0$. The lower *C*-convexity of $F(x, \cdot )$ implies

$$\begin{aligned} F\left( y,y+\dfrac{t(x_{n_k} - y)}{\Vert x_{n_k} - y \Vert } \right) \subseteq \left( 1- \dfrac{t}{\Vert x_{n_k} - y \Vert } \right) F(y,y) + \dfrac{t}{\Vert x_{n_k} - y \Vert }F(y,x_{n_k}) - C. \end{aligned}$$

It follows from $F(y,y) = \{0\}$ and $F(y,x_{n_k}) \cap (int \ C) = \varnothing$ that

$$\begin{aligned} F\left( y,y+\dfrac{t(x_{n_k} - y)}{\Vert x_{n_k} - y \Vert } \right) \cap (int \ C) = \varnothing . \end{aligned}$$

Since $y+\dfrac{t(x_{n_k} - y)}{\Vert x_{n_k} - y \Vert } \rightharpoonup y + t d_0$ and *F* is weakly lower semicontinuous at second argument, we have that $F(y,y+td_0) \cap (int \ C) = \varnothing$, and so $d_0 \in R_1$. This is a contradiction. Hence (iv) holds.

(iv) $\Rightarrow$ (ii) Let $G : K \rightarrow 2^K$ be a set-valued mapping defined by

6 $$\begin{aligned} G(y) := \{ x\in K : F(y,x) \cap (int \ C) = \varnothing \}, \quad \forall y\in K. \end{aligned}$$

We first prove that *G*(*y*) is a closed subset of *K* . Indeed, for any $x_n \in G(y)$ with $x_n \rightarrow x_0$, we have $F(y,x_n) \cap (int \ C) = \varnothing$. It follows from the weakly lower semicontinuity of $F(x,\cdot )$ that $F(y,x_0) \cap (int \ C) = \varnothing$. This shows that $x_0 \in G(y)$ and so *G*(*y*) is closed.

Next, we will show that *G* is a KKM mapping. Suppose to the contrary that there exist $\alpha _1, \alpha _2, \ldots , \alpha _n \in (0,1)$ with $\alpha _1+\alpha _2+\cdots +\alpha _n = 1 , y_1, y_2, \ldots , y_n \in K$ and $\bar{y} = \alpha _1y_1 + \alpha _2y_2 + \cdots + \alpha _ny_n \in co\{y_1, y_2,\ldots , y_n\}$ such that $\bar{y} \notin \cup _{i\in \{ 1,2,\ldots ,n \}} G(y_i)$. Then

$$\begin{aligned} F(y_i,\bar{y}) \cap (int \ C) \ne \varnothing , \quad i=1,2,\ldots, n. \end{aligned}$$

Using $$ (F_1) $$ yields

7 $$\begin{aligned} F(\bar{y},y_i) \cap (-int \ C) \ne \varnothing , \quad i=1,2,\ldots, n. \end{aligned}$$

The upper *C*-convexity of *F* implies

$$\begin{aligned} \alpha _1 F(\bar{y},y_1) + \alpha _2 F(\bar{y},y_2) + \cdots + \alpha _n F(\bar{y},y_n) \subseteq F(\bar{y},\bar{y}) + C = 0 + C \subseteq C. \end{aligned}$$

This is a contradiction with (7). Therefore, *G* is KKM mapping.

We may assume that *B* is a bounded closed convex set (otherwise, consider the closed convex hull of *B* instead of *B* ). Let $\{y_1,\ldots , y_m\}$ be finite number of points in *K* and let $M:=co(B\cup \{ y_1,y_2,\ldots ,y_m \})$. Then the reflexivity of the space *X* yields that *M* is weakly compact convex. We consider the set-valued mapping $G'$ which defined by $G'(y) := G(y)\cap M$ for all $y \in M$. Then each $G'(y)$ is a weakly compact convex subset of *M* and $G'$ is a KKM mapping. We claim that

8 $$\begin{aligned} \varnothing \ne \cap _{y\in M}G'(y) \subset B. \end{aligned}$$

By Lemma 2, the intersection in (8) is nonempty. Moreover, if there exists some $x_0 \in \cap _{y\in M}G'(y)$ but $x_0 \notin B$, then by (iv), we have $F(y,x_0)\cap (int \ C) \ne \varnothing$ for some $y\in B$. Thus, $x_0 \notin G(y)$ and so $x_0 \notin G'(y)$, which is a contradiction to the choice of $x_0$.

Let $z \in \cap _{y\in M} G(y)$. Then, by (8) we get $z \in B$, and so $z\in \cap _{i=1}^m (G(y_i)\cap B)$. This shows that the collection $\{G(y) \cap B : y \in K \}$ has finite intersection property. For each $y \in K$, it follows from the weak compactness of $G(y) \cap B$ that $\cap _{y\in K} (G(y)\cap B)$ is nonempty, which coincides with the solution set of $S^D_W(F,K)$. The proof is complete. $\square$

The following example show that Theorem 2 is applicable.

### *Example 3*

Let $X=\mathbb {R} , K=[0,+\infty ) , Y=\mathbb {R}^2 , C=\mathbb {R}^2_+ , e=(1,1)\in int\, C$. A set-valued map $F: K \times K \rightarrow 2^{\mathbb {R}^2}\backslash \{\varnothing \}$ is defined by

$$\begin{aligned} F(x,y) = \{ y-x \} \times [(y-x),(1+x)(y-x)], \quad \forall x,y\in K. \end{aligned}$$

We have that $K_\infty = [0,+\infty )$ and $C^{*0} := \left\{ (x_1,x_2)\in \mathbb {R}^2, x_1+x_2 = 1, x_1\ge 0 \text { and } x_2 \ge 0 \right\}$. It is easily seen that *F* is satisfied conditions $(F_0)$-$(F_4)$. To verify $(F_5)$ holds, we fixed $\bar{y}\in [0,+\infty )$ and consider the following set,

$$\begin{aligned} \{ x\in K:F(\bar{y},x)\cap int\,C = \varnothing \}& = \{ x\in [0,+\infty ) : x-\bar{y}\le 0 \text { or } (1+y)(x-\bar{y})\le 0 \}\\ &= \{ x\in [0,+\infty ) : x \le \bar{y} \} = [0,\bar{y}] \text { is convex set}. \end{aligned}$$

Obviously,

$$\begin{aligned} R_1 &= \{ d\in K_\infty : F(y,y+td) \cap (int \ C) = \varnothing , \ \forall y\in K, t>0 \} \\ &= \{ d\in [0,+\infty ) : td \le 0, \ \forall t >0\text { and } \forall y\in [0,+\infty ) \} = \{0\}. \end{aligned}$$

Hence, Theorem 2 concludes that $S^P_W(F,K)$ is nonempty and bounded. It follows from direct calculating that $S^P_W(F,K) = \{0\}$.

In what follow, we shall discuss the relationship between the nonemptiness and boundedness of the solution set for (GWVEP) and the solution set for (GWVEP) which *F* is composed by $\xi \in C^*$. We recall the concept of $\xi$-efficient solution set for (GWVEP) as follows.

For any fixed $\xi \in C^{*0}$, the real set-valued map $\xi (F):K\times K \rightarrow 2^\mathbb {R}\backslash \{\varnothing \}$ is defined by

9 $$\begin{aligned} \xi (F)(x,y) := \{ \xi (z) : z\in F(x,y) \}, \quad \forall x,y \in K. \end{aligned}$$

A vector $x \in K$ is called $\xi$-weak efficient solution to the (GWVEP) if

$$\begin{aligned} \inf _{z\in F(x,y)} \xi (z) \ge 0, \quad \forall y \in K, \end{aligned}$$

and $\xi$-weak efficient solution to the (DGWVEP) if

$$\begin{aligned} \sup _{z\in F(y,x)} \xi (z) \le 0, \quad \forall y \in K. \end{aligned}$$

Denote by $S^P_{\xi }(K,F)$ and $S^D_{\xi }(K,F)$ the set of all $\xi$-weak efficient solution to the (GWVEP) and (DGWVEP), respectively.

The following lemma characterizes relation between $S^P_W(K,F)$ and $S^P_{\xi }(K,F)$.

### **Lemma 7**

*Suppose that*$int \ C \ne \varnothing$*and for any*$x\in K, F(x,K) + C$*is a convex set. Then*,

$$\begin{aligned} S^P_W(K,F) = \cup _{\xi \in C^{*}\backslash \{0\}} S^P_{\xi }(K,F) = \cup _{\xi \in C^{*0}} S^P_{\xi }(K,F). \end{aligned}$$

### *Proof*

($\supseteq$) Let $x_0\in \cup _{\xi \in C^{*0}} S^P_{\xi }(K,F)$. Then there exists $\xi _0\in C^{*0}$ such that

10 $$\begin{aligned} \xi _0(z) \ge 0 \quad \text{for all}\quad y\in K \;\; \text{for all}\quad z\in F(x_0,y). \end{aligned}$$

We claim that $x_0 \in S_W^P(K,F)$. If not, then there exists $y_0 \in K$ such that

$$\begin{aligned} \xi _0(z_0) < 0 \quad \text {for some } z_0\in F(x_0,y_0). \end{aligned}$$

This is a contradiction with (10). Hence, we have desired.

($$ \subseteq $$) Let $$ x_0 \in S_W^P(K,F) $$. Then,

$$\begin{aligned} F(x_0,y) \cap (-int \ C) = \varnothing \quad \text{for all}\quad y\in K. \end{aligned}$$

This implies that

$$\begin{aligned} F(x_0,K) \cap (-int \ C) = \varnothing . \end{aligned}$$

Since *C* is a pointed convex cone, we have

$$\begin{aligned} \left( F(x_0,K) + C \right) \cap (-int \ C) = \varnothing . \end{aligned}$$

Using the separation theorem for convex sets, there exists some $\xi ' \in Y^*\backslash \{0\}$ such that

11 $$\begin{aligned} \inf \{ \xi '\left( F(x_0,y) + c : y\in K, \ c\in C \right) \} \ge \sup \{ \xi '(-c) : c\in C \}. \end{aligned}$$

From (11), we get $\xi ' \in C^*\backslash \{0\}$ and so

$$\begin{aligned} \xi ' (z) \ge 0 \quad \text{for all}\quad z\in F(x_0,y) \quad \text{for all}\quad y\in K. \end{aligned}$$

By our hypothesis, we have $C^{*0}$ is a weakly compact base for $C^*$ and we can choose $e\in int \ C$ with $\xi '(e)>0$. Setting $\xi '' = \dfrac{\xi '}{\xi '(e)}$, we then have that $\xi ''\in C^{*0}$ and

$$\begin{aligned} \xi '' (z) \ge 0 \quad \text{for all}\quad z\in F(x_0,y) \quad \text{for all}\quad y\in K. \end{aligned}$$

Hence, $x_0\in S^P_{\xi ''}(K,F) \subseteq \cup _{\xi \in C^{*0}} S^P_{\xi }(K,F)$. This completes the proof. $\square$

The following corollary give the sufficient conditions for nonemptiness and boundedness of solution set for (GWVEP) in the case of real set-valued mappings.

It follows from Theorem 2, we can derive the following corollary in the case where $F:K\times K \rightarrow 2^\mathbb {R}\backslash \{\varnothing \}$.

### **Corollary 1**

*LetKbe a nonempty closed convex subset ofXand*$F : K \times K \rightarrow 2^\mathbb R\backslash \{\varnothing \}$*be a set-valued mapping satisfying assumptions* (*F*_0_)–(*F*_4_).

*Suppose that*$int (barr(K)) \ne \varnothing$. *Then the following statements are equivalent.*

(i) *the solution set of*$S^P_W(K,F)$*is nonempty and bounded*;
(ii) *the solution set of*$S^D_W(K,F)$*is nonempty and bounded*;
(iii) $$ R=\{ d\in K_\infty : \sup _{z\in F(y,y+td)} z \le 0, \ \forall y\in K, t>0 \}=\{0\} $$;
(iv) *there exists a bounded set*$B \subset K$*such that for every*$x \in K \backslash B$, *there exists*$y \in B$*such that*$z>0$*for some*$z\in F(y,x)$.

### *Proof*

We see that *F* satisfies the assumption $(F_0)$-$(F_4)$ in Theorem 2. It is easy to verify, by $(F_2)$, that $(F_5)$ is satisfied. $\square$

By virtue of Lemma 7, one sees that the solution set for (GWVEP) can be represented by union of real set-valued $\xi (F)$ mappings. This means that the nonemptiness of $S^P_\xi (K,F)$ guarantees the existence of solution for (GWVEP). We next establish the existence theorem for $\xi$-weak efficient solution to the (GWVEP).

By the idea of linear scalarization technique, for any $\xi \in C^{*0}$, we first introduce the set

$$\begin{aligned} R^{\xi }_{1} := \left\{ d\in K_\infty : \sup _{z\in F(y,y+td)} \xi (z) \le 0, \ \forall y\in K, t>0 \right\} . \end{aligned}$$

The following lemma shows that the condition of $\cup _{\xi \in C^{*0}}R^{\xi }_{1} = \{0\}$ is weaker than $R_1 = \{0\}$.

### **Lemma 8**

$$ R_1 = \{0\} \Rightarrow \cup _{\xi \in C^{*0}}R^{\xi }_{1} = \{0\}$$.

### *Proof*

Assume that $R_1=\{0\}$. Let $d_0\in \cup _{\xi \in C^{*0}}R_{\xi }$. Then there exists $\xi _0\in C^{*0}$ and $d_0\in K_\infty$ such that for every $y\in K$ and $t>0$

12 $$\begin{aligned} \xi _0\left( z\right) \le 0 \quad \text{for all}\quad z\in F(y,y+td_0). \end{aligned}$$

We claim that for any $z\in F(y,y+td_0) , z\notin int \ C$. If not, there exists $z_0\in F(y,y+td_0)$ such that $z\in int \ C$ and so

13 $$\begin{aligned} \xi _0(z_0) > 0, \end{aligned}$$

which leads to contradiction with (12). Hence, for every $y\in K$ and $t>0$

$$\begin{aligned} F(y,y+td_0) \cap (int \ C) = \varnothing . \end{aligned}$$

By our hypothesis, $d_0=0$.

The following example shows that the inverse implication of Lemma 8 may not be true.

The following example has been changed format.

### *Example 4*

Let $X=\mathbb {R} , K=[0,+\infty ) , Y=\mathbb {R}^2 , C=\mathbb {R}^2_+ , e=(1,1)\in int \ C$. Define $F:K\times K \rightarrow 2^Y\backslash \{\varnothing \}$ by

$$\begin{aligned} F(x,y) = {\left\{ \begin{array}{ll} \{0\} \times [0,1-|y-x|], &\quad \text {if } 0 \le |y-x| \le 1, \\ {[}|y-x|-1,0] \times \{0\}, &\quad \text {if } |y-x|>1. \end{array}\right. } \end{aligned}$$

Then $$ K_\infty = [0,+\infty ) $$ and $$ C^{*0} = \{ (x_1,x_2) \in \mathbb {R}^2_+ : x_1 + x_2 = 1 \}. $$

We see that for any $y\in \mathbb {R}_+ , d\in \mathbb {R}$ and $t>0$,

$$\begin{aligned} F(y,y+td) \subseteq {\left\{ \begin{array}{ll} \{0\}\times [0,1], &\quad \text {if } 0 \le |td| \le 1, \\ {[}0,+\infty )\times \{0\}, &\quad \text {if } |td| > 1, \end{array}\right. } \end{aligned}$$

which implies that $F(y,y+td) \cap int\,C = \varnothing$ for all $y\in \mathbb {R}_+ , d\in \mathbb {R}$ and $t>0$. Hence, $R_1 = [0,+\infty )$. But, for any $\xi \in C^{*0}$, we have for any $y,d\in \mathbb {R}_+$ and $t>0$

$$\begin{aligned} \xi (z) \ge 0, \quad \text{for all}\quad x\in F(y,y+td), \end{aligned}$$

which implies that *d* must be 0 , and so $R_1^\xi = \{0\}$ for all $\xi \in C^{*0}$.

From the Corollary 1, we can obtain the following characterization corollary for $\xi$-efficient solution $S^P_{\xi }(K,F)$ and $S^D_{\xi }(K,F)$.

### **Corollary 2**

*Let*$\xi \in C^{*0}$*be any given. LetKbe a nonempty closed convex subset ofXand*$F : K \times K \rightarrow 2^Y\backslash \{\varnothing \}$*be a set-valued mapping satisfying assumptions*$(F_0) , (F_2)$-$(F_4)$*and* (v) *in Definition* 3. *Suppose that*$int (barr(K)) \ne \varnothing$. *Then the following statements are equivalent*:

(i) *the solution set of*$S^P_{\xi }(K,F)$*is nonempty and bounded*;
(ii) *the solution set of*$S^D_{\xi }(K,F)$*is nonempty and bounded*;
(iii) $$ R^{\xi }_1=\{0\} $$;
(iv) *there exists a bounded set*$B \subset K$*such that for every*$x \in K \backslash B$, *there exists*$y \in B$*such that*$\xi (z)>0$*for some*$z\in F(y,x)$.

### *Proof*

For any fixed $\xi \in C^*\backslash \{0\}$, we define $\xi (F):K\times K\rightarrow 2^{\mathbb {R}}\backslash \{\varnothing \}$ as in (9). It is not hard to check that $\xi (F)$ satisfies conditions $(F_0)$-$(F_4)$ in Corollary 1. $\square$

We now characterize the nonemptiness and boundedness of $S^P_W(K,F)$ in term of nonemptiness and boundedness of the solution set $S^P_\xi (K,F)$ for any $\xi \in C^{*0}$.

### **Theorem 3**

*LetXbe a reflexive Banach space andKbe a closed convex subset ofXwith*$int (barrK) \ne \varnothing$. *LetYbe a normed space and*$C^{*0}$*is a compact base of*$C^{*}$. *Suppose that*$F : K \times K \rightarrow 2^Y\backslash \{\varnothing \}$*is a set-valued mapping satisfying assumptionsF*_0_ (*F*_2_)–(*F*_4_) *and (v) in Definition* 3.

*Then*$S_W^P(K,F)$*is nonempty and bounded if and only if for any*$\xi \in C^{*0} , S^P_{\xi }(K,F)$*is nonempty and bounded*.

### *Proof*

Suppose that for any $\xi \in C^{*0} , S^P_{\xi }(K,F)$ is nonempty and bounded. Then by Corollary 2, $R^{\xi }_{1}= \{0\}$. We claim that $S^P_W(K,F)$ is nonempty and bounded. The nonemptiness of $S^P_W(K,F)$ is obvious, because of $S^P_{\xi }(K,F)\subset S^P_W(K,F)$. We only need to show that $S^P_W(K,F)$ is bounded. If not, there exists a sequence $x_n \in S^P_W(K,F)$ such that $\Vert x_n \Vert \rightarrow +\infty$. Since $x_n \in S^P_W(K,F)$, we then have

$$\begin{aligned} F(x_n,y) \cap (-int \ C) = \varnothing , \quad \text{for all}\quad y\in K. \end{aligned}$$

Thus, for every $z_n\in F(x_n,y) , z_n\notin -int \ C$. Then there exists $\xi _n\in C^{*0}$ such that

$$\begin{aligned} \xi _n(z_n) \ge 0, \quad \text{for all}\quad z\in F(x_n,y), \;\;\text{for all}\quad y\in K. \end{aligned}$$

By the $\xi$-pseudomonotonicity of *F* , we have

14 $$\begin{aligned} \xi _n(z'_n) \le 0, \quad \text{for all}\quad z'\in F(y,x_n), \;\;\text{for all}\quad y\in K \end{aligned}$$

Since $C^{*0}$ is compact, without loss of generality, we can assume that $\xi _n \rightarrow \xi _0 \in C^{*0}$. For any fixed $y\in K$ and $t>0$, without loss of generality, we may take a subsequence $\{x_{n_k}\}$ of $\{x_n\}$ such that

$$\begin{aligned} \dfrac{t}{\Vert x_{n_k} - y\Vert } \in (0,1) \quad\text{and}\quad w-\lim \limits _{k\rightarrow +\infty } \dfrac{x_{n_k} - y}{\Vert x_{n_k} - y \Vert } = w-\lim \limits _{k\rightarrow +\infty } \dfrac{x_{n_k}}{\Vert x_{n_k}\Vert } = d_0\in K_\infty . \end{aligned}$$

By Lemma 3, $d_0\ne 0$. Upper *C*-convexity of *F* implies

$$\begin{aligned} \left( 1- \dfrac{1}{\Vert x_{n_k} - y \Vert } \right) F(y,y) + \dfrac{t}{\Vert x_{n_k} - y \Vert }F(y,x_{n_k}) \subseteq F\left( y,y+\dfrac{t(x_{n_k} - y)}{\Vert x_{n_k} - y \Vert } \right) + C \end{aligned}$$

It follows from $F(y,y) = \{0\}$ and (14) that for any $\xi _n$,

$$\begin{aligned} \xi _n\left( F\left( y,y+\dfrac{t(x_{n_k} - y)}{\Vert x_{n_k} - y \Vert } \right) \right)&\le  \left( 1- \dfrac{1}{\Vert x_{n_k} - y \Vert } \right) \xi _n\left( F(y,y)\right) + \dfrac{t}{\Vert x_{n_k} - y \Vert }\xi _n\left( F(t,x_{n_k})\right) \\ & \le 0. \end{aligned}$$

Since *F* is weakly lower semicontinuous at second variable and $\xi _n \rightarrow \xi _0$, we have

$$\begin{aligned} \xi _0(F(y,y+td_0)) \le 0. \end{aligned}$$

This implies that $0 \ne d_0\in R_1^{\xi }$, which is a contradiction.

Conversely, we assume that $S^P_W(K,F)$ is nonempty and bounded. We claim that $S^P_{\xi }(K,F)$ is nonempty and bounded for all $\xi \in C^{*0}$. We consider the set $A\subseteq C^{*0}$ as follows.

$$\begin{aligned} A := \{ \xi \in C^{*0} : S^P_{\xi }(K,F) \text { is nonempty and bounded } \}. \end{aligned}$$

Clearly, *A* is nonempty. Firstly, we claim that *A* is open subset in $C^{*0}$. If not, there exists $\xi _0\in A$ and a sequence $\xi _n\in C^{*0}$ with $\xi _n \rightarrow \xi _0$ such that $\xi _n \notin A$. This implies that $R^{\xi _n}_1 \ne \{0\}$. Then there exists $d_n\in R^{\xi _n}_1$ such that $\Vert d_n\Vert =1$. Since $C^{*0}$ is compact and $\Vert d_n\Vert =1$, without loss of generality, we may assume that $d_n \rightharpoonup d_0 \in K_\infty \backslash \{0\}$. Since $d_n\in R^{\xi _n}_{1}$, we have

$$\begin{aligned} \xi _n(z') \le 0 \quad \text{for all}\quad z'\in F(y,y + t d_n) \;\; \text{for all}\quad y\in K. \end{aligned}$$

Since *F* is weakly lower semicontinuous at second variable and $\xi _n \rightarrow \xi _0$, we have

$$\begin{aligned} \xi _0(z') \le 0 \quad \text{for all}\quad z'\in F(y,y+td_0) \;\; \text{for all}\quad y\in K. \end{aligned}$$

Thus $0 \ne d_0\in R_{\xi _0}$. This implies that $S^P_{\xi _0}(K,F)$ is not nonempty and bounded, which leads to a contradiction with $\xi _0\in A$. Hence *A* is an open subset of $C^{*0}$.

Finally, we claim that *A* is a closed subset of $C^{*0}$. Let $\xi _n\in A$ with $\xi _n \rightarrow \xi _0$. In view of $\xi _n\in A$, we have $S_{\xi _n}(K,F)$ is nonempty and bounded. Let $x_n\in S^P_{\xi _n}(K,F)$. Whereas $S^P_{\xi _n}(K,F)\subset S^P_W(K,F)$ and $S^P_W(K,F)$ is bounded, $\{x_n\}$ is also. We may assume that $x_n \rightharpoonup x_0 \in K$. Since $x_n \in S_{\xi _n}(K,F)$, then we have

$$\begin{aligned} \xi _n(z) \ge 0 \quad \text{for all}\quad z\in F(x_n,y) \;\; \text{for all}\quad y\in K. \end{aligned}$$

By $\xi$-pseudomonotonicity of *F* , we get

$$\begin{aligned} \xi _n(z') \le 0, \quad \text{for all}\quad z'\in F(y,x_n), \;\;\text{for all}\quad y\in K. \end{aligned}$$

Since *F* is weakly lower semicontinuous at the second variable, letting $n\rightarrow \infty$

$$\begin{aligned} \xi _0(z') \le 0, \quad \text{for all}\quad z'\in F(y,x_0), \;\; \text{for all}\quad y\in K. \end{aligned}$$

Hence, $x_0 \in S^D_{\xi _0}(K,F)$. Thanks to Corollary 2, we get that $x_0\in S^P_{\xi _0}(K,F)$. The boundedness of $S^P_W(K,F)$ implies $S^P_{\xi _0}(K,F)$ is also. This means that $\xi _0\in A$ and so *A* is closed. Since the base $C^{*0}$ of $C^*$ is connected, we have *A* must be $C^{*0}$. $\square$

### **Theorem 4**

*LetXbe a reflexive Banach space andKbe a closed convex subset ofXwith*$int (barrK) \ne \varnothing$. *LetYbe a normed space and*$C^{*0}$*is a compact base of*$C^{*}$. *Suppose that*$F : K \times K \rightarrow 2^Y\backslash \{\varnothing \}$*is a set-valued mapping satisfying assumptions*$(F_0) , (F_2)$–$(F_4)$*and (v) in Definition* 3. *Then the following statements are equivalent*.

(i) $S^P_W(K,F)$*is nonempty and bounded*;
(ii) *For every*$\xi \in C^{*0} , S^P_{\xi }(K,F)$*is nonempty and bounded*;
(iii) $$ \cup _{\xi \in C^{*0} } R^{\xi }_{1} = \{0\} $$.

### *Remark 3*

Theorem 4 generalize Theorem 2, in the following three cases:

(i) Condition $(F_1)$ is relaxed to the condition $(F^{\xi }_1)$.
(ii) Recession cone $R_1=\{0\}$ is relaxed to the condition $\cup _{\xi \in C^{*0}}R_1^\xi =\{0\}$.
(iii) Condition $(F_5)$ is omitted.

The following example show that Theorem 4 is applicable.

### *Example 5*

Let $X=\mathbb {R} , K=[0,+\infty ) , Y=\mathbb {R}^2 , C=\mathbb {R}^2_+ , e=(1,1)\in int\, C$. A set-valued map $F: K \times K \rightarrow 2^{\mathbb {R}^2}\backslash \{\varnothing \}$ is defined by

$$\begin{aligned} F(x,y) = \{ (y-x) \} \times [(y-x),(e^{(y-x)}-1)+(y-x)] \end{aligned}$$

Then, clearly $$ (F_0) ,$$ F_2_–F_4_ and (v) in Definition 3 are satisfied. For any $$ \xi \in C^{*0} $$, we consider

$$\begin{aligned} R_1^{\xi } & = \left\{ d\in K_\infty : \sup _{z\in \left( F(y,y+td)\right) } \xi (z) \le 0, \ \forall y\in K, t>0 \right\} \\ & = \left\{ d\in [0,+\infty ) : \xi (z) \le 0, \forall z\in \{td\}\times [td,(e^{td}-1)+td] \text { and } \ \forall y\in K, t>0 \right\} = \{0\}. \end{aligned}$$

It follows from Theorem 4 that, $S^P_W(K,F)$ is nonempty and bounded.

## Stability analysis

In this section, we shall establish the stability theorem of solution set for (GWVEP) when the mapping *F* and the domain set *K* are perturbed by different parameters.

We first recall some important notions . Let $(\Lambda ,d_{\Lambda })$ and $(M, d_M)$ be two metric spaces. Let $K(\lambda )$ be perturbed by a parameter $\lambda$, which varies over $(\Lambda , d_{\Lambda })$, that is, $K : \Lambda \rightarrow 2^X$ is a set-valued mapping with nonempty, closed, and convex values. Let *F* be perturbed by a parameter $\mu$, which varies over $(M,d_{M})$, that is, $F : K \times K \times M \rightarrow 2^Y\backslash \{ \varnothing \}$ is a parametric set-valued mapping.

Consider the parametric generalized weak vector equilibrium problems, denoted by (PGWVEP), which consists in finding $\bar{x} \in K(\lambda )$ such that

PGWVEP $$\begin{aligned} F(\bar{x},y,\mu ) \cap (- int \ C) \ne \varnothing \quad \forall y\in K(\lambda ). \end{aligned}$$

Denote by $S^P_W(\lambda ,\mu )$ the set of all weak efficient solution to the (PGWVEP).

Let $\{A_n\}$ be a sequence of nonempty subset of *X* . We define

$$\begin{aligned} \limsup _{n\rightarrow +\infty } A_n := \left\{ x\in X : \exists \{n_k\}, x_{n_k}\in A_{n_k} \text { such that } x_{n_k} \rightarrow x \right\} . \end{aligned}$$

We say that the sequence $\{A_n\}$ is of upper convergence in the sense of Painle$\acute{\mathrm{v}}$e–Kuratowski (P.K. convergence) Durea (2007) to *A* if $\limsup _{n\rightarrow +\infty } A_n \subseteq A$.

The following theorem shows that under suitable situation, there exists a neighborhood $N(\lambda _0)\times N(\mu _0)$ of $(\lambda _0,\mu _0)$ such that $S^P_W(\lambda ,\mu )$ P.K. convergence to $S^P_W(\lambda _0,\mu _0)$ in the neighborhood $N(\lambda _0)\times N(\mu _0)$.

### **Theorem 5**

*LetXbe a real reflexive Banach space andKbe a closed convex subset ofXwith*$int (barrK) \ne \varnothing$. *LetYbe a normed space and*$C^{*0}$*is a compact base of*$C^{*}$. *Suppose thatFsatisfies the following conditions*:

(I) $K(\cdot )$*is continuous on*$\Lambda$*and*$int (\ barr \ K(\lambda _0)) \ne \varnothing$, *for all*$\lambda \in \Lambda$*and has nonempty closed convex valued*.
(II) *For any*$\lambda \in \Lambda$*and*$x\in K(\lambda ) , F(x,x,\mu ) = \{0\}$.
(III) *For any*$\lambda \in \Lambda$*and*$\mu \in M , F(\cdot ,\cdot ,\mu )$*is*$\xi$-*pseudomonotone on*$K(\lambda )$*w.r.t*. $C^{*0}$.
(IV) *For any*$\mu \in M$*and*$x\in K(\mu ) , F(x,\cdot ,\mu )$*isC*-*convex*.
(V) *For any*$\lambda \in \Lambda$*and*$\mu \in M , F(\cdot ,\cdot ,\cdot )$*is continuous on*$K(\lambda )\times K(\lambda ) \times M$.

*If*$S^P_W(\lambda _0,\mu _0)$*is nonempty and bounded, then the following statements hold*.

(i) there exists a neighborhood $N(\lambda _0)\times N(\mu _0)$ such that $S_W^P(\lambda ,\mu )$ has a nonempty and bounded for all $(\lambda ,\mu )\in N(\lambda _0)\times N(\mu _0)$.
(ii) $$ \limsup _{(\lambda ,\mu )\rightarrow (\lambda _0,\mu _0)} S^P_W(\lambda ,\mu ) \subseteq S^P_W(\lambda _0,\mu _0) $$.

### *Proof*

(i) We claim that there exists a neighborhood $N(\lambda _0)\times N(\mu _0)$ of $(\lambda _0,\mu _0)$ such that for any $(\lambda ,\mu )\in N(\lambda _0)\times N(\mu _0)$ and $\xi \in C^{*0}$

$$\begin{aligned} R_1^{\xi }(\lambda ,\mu ) := \left\{ d\in K(\lambda )_\infty : \sup _{z\in \left( F(y,y+td,\mu )\right) } \xi (z) \le 0, \ \forall y\in K, t>0 \right\} =\{0\}. \end{aligned}$$

If not, there exists $(\lambda _n, \mu _n)\in \Lambda \times M$ with $(\lambda _n, \mu _n)\rightarrow (\lambda _0,\mu _0)$ and $\xi '\in C^{*0}$ such that $R^{\xi '}_{1} (\lambda _n,\mu _n) \ne \{0\}$.

Since *K* is lower semicontinuous at $\lambda _0$, for any $y \in K(\lambda _0)$, we have $y_n \in K(\lambda _n)$ such that $y_n \rightarrow y$. Together with $\mu _n \rightarrow \mu _0$, we have $(y_n, \mu _n)\rightarrow (y,\mu _0)$. Thus, we can select a sequence $\{d_n\}$ such that

15 $$\begin{aligned} d_n\in K(\lambda _n)_\infty \text { and } \sup _{z\in F(y_n,y_n+td_n,\mu _n)} \xi ' \left( z \right) \le 0, \quad \forall y\in K(\lambda _n), t>0. \end{aligned}$$

with $\Vert d_n\Vert =1$ for all $n=1,2,\ldots$. Since $\{d_n\}$ is a bounded sequence in a reflexive Banach space *X* we can assume that $d_n \rightharpoonup d_0$. It follows from Lemma 4 that $d_0 \ne 0$. We claim that $d_0\in K(\lambda _0)_\infty$. Since *K* is upper semicontinuous at $\lambda _0$ and $d_n\in K(\lambda _n)_\infty$, by Lemma 5, we have that $d_n\in K(\lambda _0)_\infty$, for all sufficiently large *n* . By the closure of $K(\lambda _0)_\infty$, we have $d_0 \in K(\lambda _0)_\infty$. Notice that the continuity assumption of *F* , taking the limit in (15), we have

$$\begin{aligned} \sup _{z\in F(y,y+td_0,\mu _0)}\xi ' \left( z\right) \le 0, \end{aligned}$$

which implies that $0 \ne d_0 \in R_1^{\xi '}(\lambda _0,\mu _0)$. This is a contradiction with $S^P_W(\lambda _0,\mu _0) \ne \varnothing$, so we have the claim.

(ii) We want to show that for any $(\lambda ,\mu )\rightarrow (\lambda _0,\mu _0)$,

$$\begin{aligned} \limsup _{(\lambda ,\mu )\rightarrow (\lambda _0,\mu _0)} S^P_W(\lambda ,\mu )\subseteq S^P_W(\lambda _0,\mu _0). \end{aligned}$$

Let $\bar{x}\in \limsup _{(\lambda ,\mu )\rightarrow (\lambda _0,\mu _0)} S^P_W(\lambda ,\mu )$. Then there exits a sequence $x_{n_k}\in S^P_W(\lambda _{n_k},\mu _{n_k})$ such that $x_{n_k} \rightarrow \bar{x}$ as $k\rightarrow \infty$. Since *K* is upper semicontinuous at $\lambda _0$, for sufficiently large *n* we get that

$$\begin{aligned} K(\lambda _n) \subseteq K(\lambda _0) + \dfrac{1}{n}B, \end{aligned}$$

where *B* is a closed unit ball. By virtue of $x_{n_k}\in K(\lambda _{n_k})$, we get that

$$\begin{aligned} d(x_{n_k},K(\lambda _0)) \le \dfrac{1}{n_k} \rightarrow 0. \end{aligned}$$

It follows from $K(\lambda _0)$ is closed and $x_{n_k}\rightarrow \bar{x}$ that $\bar{x}\in K(\lambda _0)$.

Since *K* is lower semicontinuous at $\lambda _0$, for any $y\in K(\lambda _0)$ there exists $y_{n_k}\in K(\lambda _{n_k})$ with $y_{n_k} \rightarrow y$. By our hypothesis, we get

$$\begin{aligned} F(x_{n_k},y_{n_k},\mu _{n_k})\cap (-int\,C) = \varnothing . \end{aligned}$$

Continuity of *F* implies

$$\begin{aligned} F(\bar{x},y,\mu _0)\cap (-int\,C) = \varnothing . \end{aligned}$$

Since the latest inequality holds for all $y\in K(\lambda _0)$. Hence, $\bar{x}\in S^P_W(\lambda _0,\mu _0)$. $\square$

## Conclusions

In this paper, some characterizations of nonemptiness and boundedness of solution sets for generalized weak vector equilibrium problems are established in a reflexive Banach space. By using the linear scalarization method, we give a sufficient and necessary condition for the nonemptiness and boundedness of $S^P_W(K,F)$ in term of nonemptiness and boundedness of the solution set $S^P_\xi (K,F)$ for any $\xi \in C^{*0}$. As application, we discuss the stability result for the solution set to (PGWVEP) in the sense of Painlevé–Kuratowski upper convergence of set.

## References

[CR1] Adly S, Ernst E, Théra M (2004). Well-positioned closed convex sets and well-positioned closed convex functions. J Glob Optim.

[CR2] Ansari QH, Konnov IV, Yao JC (2001). On generalized vector equilibrium problems. Nonlinear Anal.

[CR3] Ansari QH, Konnov IV, Yao JC (2001). Existence of a solution and variational principles for vector equilibrium problems. J Optim Theory Appl.

[CR4] Ansari QH, Siddiqi AH, Wu SY (2001). Existence and duality of generalized vector equilibrium problems. J Math Anal Appl.

[CR5] Ansari QH, Konnov IV, Yao JC (2002). Characterizations of solutions for vector equilibrium problems. J Optim Theory Appl.

[CR6] Ansari QH, Flores-Bazán F (2006). Recession methods for generalized vector equilibrium problems. J Math Anal Appl.

[CR7] Aubin JP, Ekeland I (1984). Applied nonlinear analysis.

[CR8] Chen B, Gong XH, Yuan SM (2008). Connectedness and compactness of weak efficient solutions for set-valued vector equilibrium problems. J Inequal Appl.

[CR9] Chen CR, Li SJ, Teo KL (2009). Solution semicontinuity of parametric generalized vector equilibrium problems. J Glob Optim.

[CR10] Durea M (2007). On the existence and stability of approximate solutions of perturbed vector equilibrium problems. J Math Anal Appl.

[CR11] Fan K (1984). Some properties of convex sets related to fixed point theorems. Math Ann.

[CR12] Fang YP, Huang NJ (2007). Equivalence of equilibrium problems and least element problems. J Optim Theory Appl.

[CR13] Fan JH, Zhong RY (2008). Stability analysis for variational inequality in reflexive Banach spaces. Nonlinear Anal.

[CR14] Ferro F (1989). A minimax theorem for vector-valued function. J Optim Theory Appl.

[CR15] Flores-Bazán F (2000). Existence theorems for generalized noncoercive equilibrium problems: the quasiconvex case. SIAM J Optim.

[CR16] Flores-Bazán F, Flores-Bazán F (2003). Vector equilibrium problems under asymptotic analysis. J Glob Optim.

[CR17] Gong XH (2008). Continuity of the solution set to parametric weak vector equilibrium problems. J Optim Theory Appl.

[CR18] Huang XX, Fang YP, Yang XQ (2014). Characterizing the nonemptiness and compactness of the solution set of a vector variational inequality by scalarization. J Optim Theory Appl.

[CR19] Li SJ, Li XB (2011). Hölder continuity of solutions to parametric weak generalized Ky Fan inequality. J Optim Theory Appl.

[CR20] Sadeqi I, Alizadeh CG (2011). Existence solutions of generalized vector equilibrium problems in reflexive Banach spaces. Nonlinear Anal.

[CR21] Xu YD, Li SJ (2013). On the lower semicontinuity of the solution mappings to a parametric generalized strong vector equilibrium problem. Positivity.

[CR22] Zhong RY, Huang NJ, Cho YJ (2011). Boundedness and nonemptiness of solution sets for set-valued vector equilibrium problems with application. J Inequal Appl.

